# fNIRS‐based evaluation of the impact of SARS‐CoV‐2 infection central auditory processing

**DOI:** 10.1002/brb3.3303

**Published:** 2023-10-31

**Authors:** Handan Yaman, Oğuz Yılmaz, Lütfü Hanoğlu, Yıldırım Bayazıt

**Affiliations:** ^1^ Department of Audiology Istanbul Medipol University Mega Hospital Istanbul Turkiye; ^2^ Department of Audiology, Faculty of Health Sciences Istanbul Medipol University Istanbul Turkiye; ^3^ Functional Imaging and Cognitive‐Affective Neuroscience Lab (fINCAN), Research Institute for Health Sciences and Technologies (SABITA) Istanbul Medipol University Istanbul Turkiye; ^4^ Department of Neurology Istanbul Medipol University, Medipol Mega Hospital Istanbul Turkiye; ^5^ Department of ENT, Gaziosmanpaşa Hospital Istanbul Yeni Yüzyıl University Istanbul Turkiye

**Keywords:** auditory attention, fNIRS, oddball paradigm, SARS‐CoV‐2

## Abstract

**Objectives:**

Coronavirus disease‐2019 due to SARS‐CoV‐2 infection has been associated with neurological and neuropsychiatric illnesses as well as auditory system problems. In this study, we aimed to evaluate the impact of SARS‐CoV‐2 infection on the central auditory system by assessing the hemodynamic activation changes using functional near‐infrared spectroscopy (fNIRS).

**Methods:**

Three participants who had SARS‐CoV‐2 infection (study group) and four participants who had no SARS‐CoV‐2 infection (control group) were included in the study. During the auditory oddball task in which two different frequencies of tonal stimulation were presented at 80 dB HL, the participants were asked to pay attention to the rare tonal stimulation and mentally count these target stimuli throughout the task. During this task, oxygenated hemodynamic response functions were evaluated with fNIRS.

**Results:**

Significantly increased oxygenated hemodynamic responses were observed in both groups during the task (*p* < .05), which was significantly higher in the study group (*p* < .05). Significantly more HbO activation was observed in the vmPFC, superior temporal gyrus, and medial temporal gyrus in the study group compared to controls (*p* < .05). Significantly higher hemodynamic activation was observed in the right hemisphere in both groups, which was significantly higher in the study group (*p* < .05).

**Conclusion:**

SARS‐CoV‐2 infections may impact on central auditory processing or auditory attention due to changes in oxyhemoglobin levels in the frontal and temporal brain regions. It seems that SARS‐CoV‐2 infection is associated with an additional load on neural activity, and difficulties in focusing in auditory attention, following speech and hearing in noise as well as increased effort to perceive auditory cues.

## Graphical abstract

In this study, we aimed to evaluate the impact of SARS‐CoV‐2 infection on the central auditory system by assessing the hemodynamic activation changes using functional near‐infrared spectroscopy (fNIRS). It seems that SARS‐CoV‐2 infection is associated with an additional load on neural activity, and difficulties in focusing in auditory attention, following speech and hearing in noise as well as increased effort to perceive auditory cues.

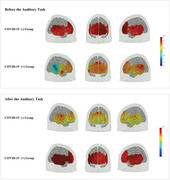

## 1 INTRODUCTION

The novel type of coronavirus due to SARS‐CoV‐2 infection has been one of the major public health problems in worldwide. In the COVID‐19 pandemic, almost one third of the asymptomatic and the majority of hospitalized cases have experienced post‐COVID‐19 problems at different severities (Huang et al., 2021; Tenforde et al., 2022). The “post‐COVID‐19 state” moniker has been proposed by the World Health Organization (WHO) for fatigue and cognitive impairment, and “post‐acute sequelae of COVID‐19” along with other persistent neuropsychiatric and physical symptoms (Soriano et al., 2022). Fatigue and cognitive impairment have been consistently reported to be some of the most common and debilitating features of the post‐COVID‐19 states (Davis et al., 2021; Marshall, 2020; Patient Led Research Collaborative 2022).

It has been proposed that the subjects recovering from SARS‐CoV‐2 infection could not focus on and process information temporarily or permanently (Carod‐Artal, 2021; Ceban et al., 2022). The consequences of this situation might be difficulty in concentrating and focusing, weakness in following and understanding speech, memory loss, and weakness in listening skills. Similar conditions may also be observed in people with hearing loss or auditory processing disorder, mild cognitive impairment, or executive dysfunction (Ceban et al., 2022; Hampshire et al., 2021). Although the previous studies reported that the auditory systems of people with SARS‐CoV‐2 infection have been affected, these studies mainly focused on the peripheral auditory system (Jafari et al., 2022; Mustafa, 2020; Sriwijitalai & Wiwanitkit, 2020; Trecca et al., 2020). However, the impact of SARS‐CoV‐2 infection on central auditory processing remains unclear.

Near‐infrared spectroscopy (NIRS) is a noninvasive neuroimaging technique that measures changes in oxygenation and cerebral blood volume in the brain. The hemodynamic signal corresponding to cerebral blood oxygenation changes are obtained by measuring the absorption of near‐infrared light through extra‐cerebral and cerebral tissue (Strait & Scheutz, 2014). It has been used to evaluate the central auditory system in various contexts, demonstrating its importance in understanding auditory function and dysfunction. In the literature, NIRS plays an important role in the evaluation of the central auditory system and has been observed to provide noninvasive and valuable information about auditory function, plasticity, and dysfunction (Calmels et al., 2022; Calmels et al., 2022; Zaramella et al., 2001). In addition, the functional NIRS method (fNIRS) is frequently used during tasks such as the oddball paradigm that requires attention to a specific target in repetitive stimulus sequences. The relationship between the oddball task and fNIRS is primarily focused on studying event‐related hemodynamic responses in the brain during this cognitive task. The oddball task is a widely used paradigm in cognitive neuroscience, which involves presenting a series of stimuli, with occasional “oddball” stimuli that differ from the majority of the stimuli. This task elicits specific neural responses which is associated with attention and cognitive processing (McLinden et al., 2023).

The Montreal Cognitive Assessment (MoCA) evaluates various cognitive domains, such as attention and concentration, executive functions, memory, language, visuoconstructional skills, and orientation (Kang et al., 2018). This comprehensive assessment provides a more holistic understanding of an individual's cognitive abilities. In our study, the MoCA test was applied to evaluate the cognitive abilities of the participants in both groups and eliminate the possibility of an underlying cognitive impairment. It consists of 30 points, and a score of 26 or above is considered normal (Mahendran et al., 2015).

In this study, we hypothesized that the subjects who had SARS‐CoV‐2 infection might have oxygenation differences in the brain regions associated with hearing and attention during the auditory cognitive paradigm. In order to test this hypothesis, hemodynamic activation changes of the frontal and bilateral temporal brain regions were assessed using NIRS during the auditory oddball paradigm.

## 2 MATERIALS AND METHODS

This study was approved by the ethical committee of Istanbul Medipol University (No: E‐10840098‐772.02‐775).

### 2.1 Participants

The study group (COVID‐19 (+)) comprised three subjects who had a history of SARS‐CoV‐2 infection 113–403 (mean, 286) days prior to this study. The subjects had positive polymerase chain reaction (PCR) results from the nasal and pharyngeal swabs, and temporary anosmia and taste disorders. They had no pulmonary involvement or dyspnea necessitating respiratory support. Their ages ranged from 23 to 25 (mean, 24.3) years.

The control group (COVID‐19 (−)) comprised four subjects who had no history of SARS‐CoV‐2 infection, positive PCR result or known contact with infected subjects. Their ages ranged from 23 to 46 (mean, 30) years.

The study and control groups were comparable regarding their ages, genders, and educational status. None of the subjects had any neurological or auditory complaints when this study was performed.

### 2.2 Experimental design

We adopted an auditory‐based oddball task that measures of attention. In the oddball paradigm, two standard stimuli (target and nontarget) were used. This paradigm included a total of 120 stimuli; 80 nontargets and 40 targets. The target and nontarget stimuli were same with an 80 dB HL sound intensity. The nontarget stimulus frequency was 1500 Hz, and the target stimulus frequency was 1650 Hz (10% more than the nontarget frequency).

**FIGURE 1:**
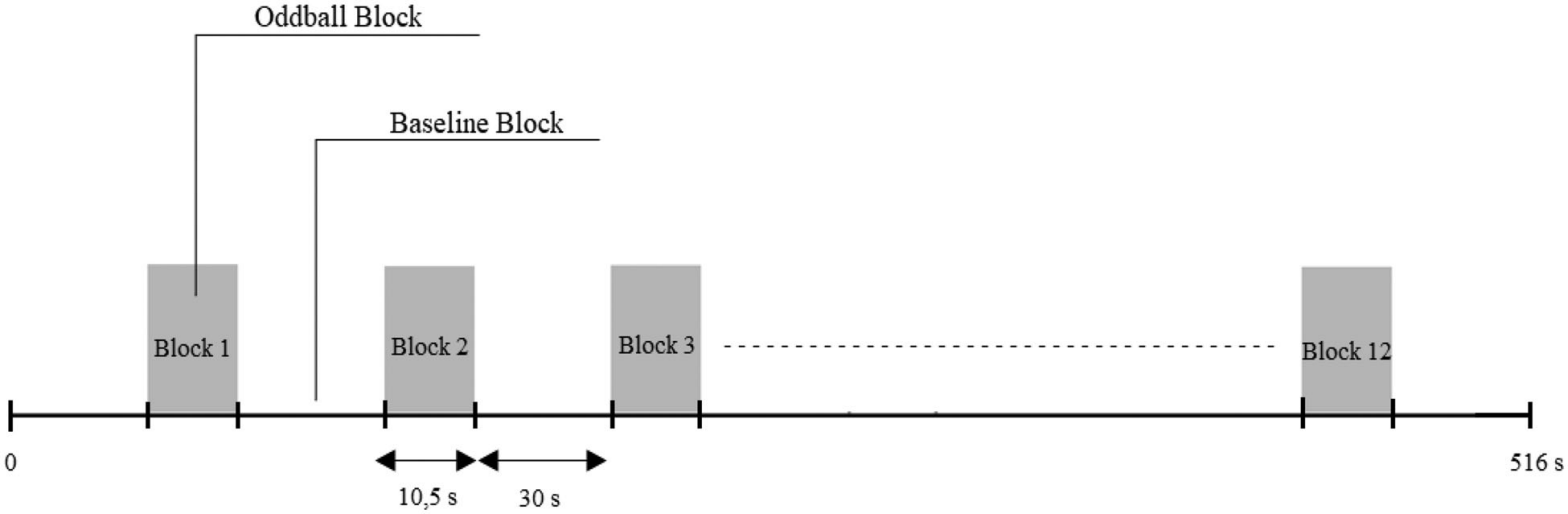
Oddball paradigm design.

The auditory stimuli were provided by positioning a pair of loudspeakers 1 m away from the subjects. The duration of the stimuli was 150 @@milliseconds (ms). The interval between stimuli was randomly presented as 800–1000 ms. The rise and fall time of the stimuli was 5 ms. The participants counted the target stimuli mentally during the paradigm. At the end of each experiment, the number of target stimuli counted by the subject was noted (Figure 1).

### 2.3 Hemodynamic recording

**FIGURE 2:**
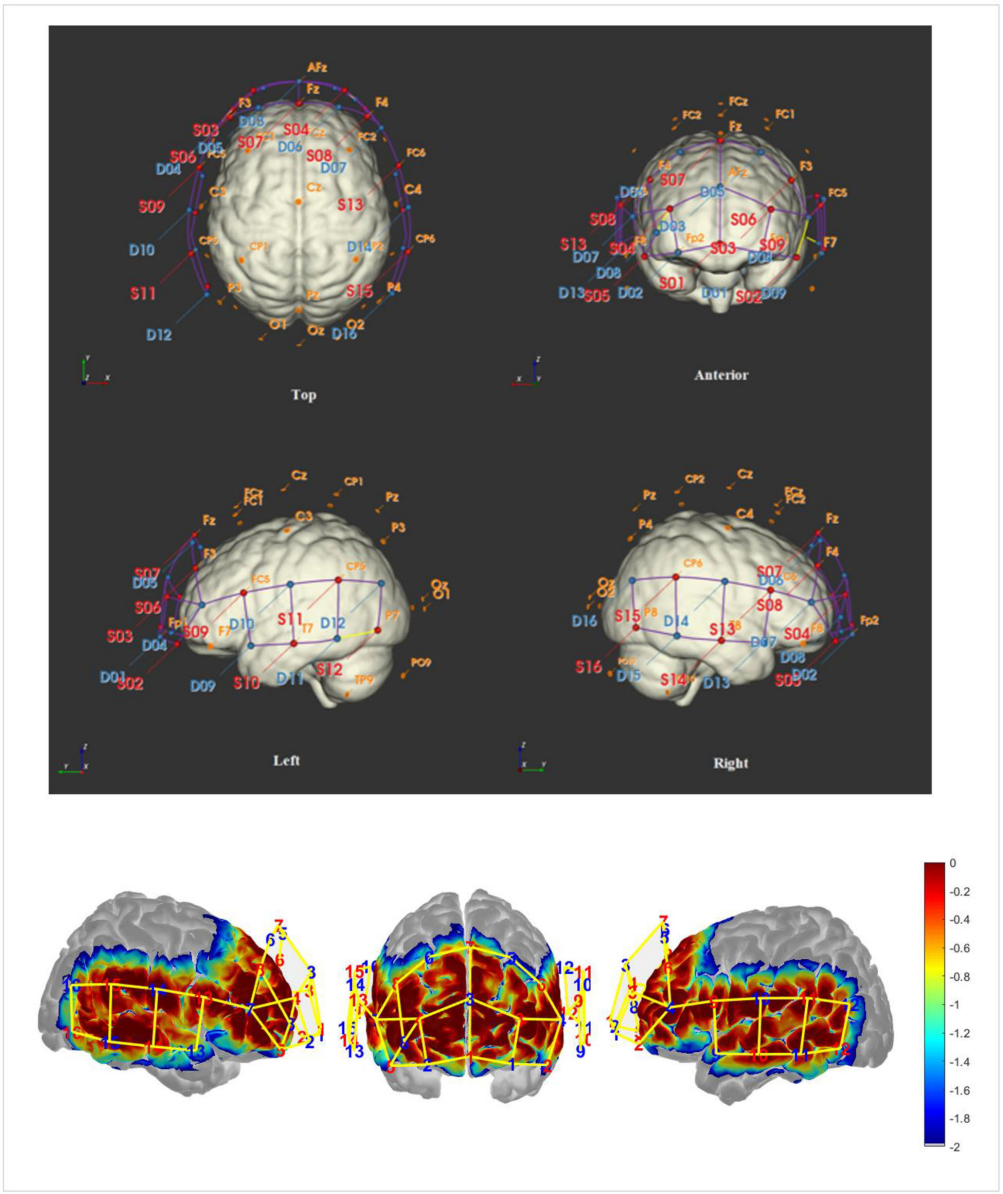
Functional near‐infrared spectroscopy (fNIRS) channels configuration and photon sensitivity profile. Each red number corresponds to a light source, whereas each blue number represents a detector.

NIRSCout extended (NIRx Medical Technologies, LLC.) device was used for the fNIRS measurements of the participants. Optical data are based on the modified Beer‐Lambert law. (Cope et al., 1988). According to the EEG 10‐10 system, 45 NIRS channels were created. Eight sources (Fpz, AF7, AF3, AF4, AF8, F3, Fz, and F4) and eight detectors (Fp1, Fp2, AFz, AF6, F1, F2, F5, and F6) located on the frontal cortex, whereas eight sources (FC5, FC6, T7, T8, CP5, CP6, P7, and P8) and eight detectors (FT7, FT8, C5, C6, TP7, TP8, P5, and P6) located on the bilateral temporal cortex (Figure 2). Detailed information about fNIRS channels is shown in Table 1.

**TABLE 1:** Brain regions to which functional near‐infrared spectroscopy (fNIRS) channels correspond.

Channels	Channel coordinates (MNI)	Label name	Channels	Channel coordinates (MNI)	Label name
**CH 1**	−10 63 −1	**L** vmPFC	**CH 24**	−55 32 20	**L** IFG
**CH 2**	14 69 −4	**R** vmPFC	**CH 25**	−50 10 10	**L** IFG
**CH 3**	2 53 10	mPFC	**CH 26**	−41 7 24	**L** IFG
**CH 4**	−30 64 −4	**L** vmPFC	**CH 27**	−40 −6 −5	**L** STG
**CH 5**	−33 46 1	**L** dlPFC	**CH 28**	−48 −14 9	**L** STG
**CH 6**	−23 68 4	**L** vmPFC	**CH 29**	−49 −27 −7	**L** MTG
**CH 7**	−12 67 21	mPFC	**CH 30**	−48 −22 27	**L** RO
**CH 8**	−25 46 8	**L** dlPFC	**CH 31**	−54 −39 13	**L** STG
**CH 9**	25 54 4	**R** vmPFC	**CH 32**	−51 −50 27	**L** SMG
**CH 10**	16 70 25	mPFC	**CH 33**	−46 −46 −5	**L** MTG
**CH 11**	40 54 10	**R** dlPFC	**CH 34**	−47 −57 15	**L** MTG
**CH 12**	34 65 10	**R** dlPFC	**CH 35**	54 30 18	**R** IFG
**CH 13**	27 53 0	**R** vmPFC	**CH 36**	59 12 8	**R** IFG
**CH 14**	51 53 1	**R** dlPFC	**CH 37**	56 7 22	**R** IFG
**CH 15**	42 59 0	**R** dlPFC	**CH 38**	56 1 −6	**R** STG
**CH 16**	−32 34 16	**L** dlPFC	**CH 39**	63 −10 8	**R** STG
**CH 17**	−14 37 27	**L** dlPFC	**CH 40**	53 −26 −8	**R** MTG
**CH 18**	2 43 24	mPFC	**CH 41**	60 −21 26	**R** SMG
**CH 19**	−2 36 33	mPFC	**CH 42**	65 −40 13	**R** STG
**CH 20**	14 52 51	mPFC	**CH 43**	58 −49 29	**R** SMG
**CH 21**	29 49 40	**R** dlPFC	**CH 44**	60 −47 −2	**R** MTG
**CH 22**	43 37 19	**R** dlPFC	**CH 45**	63 −62 16	**R** MTG
**CH 23**	32 49 11	**R** dlPFC			

Abbreviations: CH, channel; dlPFC, dorsolateral prefrontal cortex; IFG, inferior frontal gyrus; L, left; mPFC, medial prefrontal cortex; MTG, medial temporal gyrus; R, right; RO, rolandic operculum; SMG, supramarginal gyrus; STG, superior temporal gyrus; vmPFC, ventromedial prefrontal cortex.

To determine the anatomical locations of NIRS channels, the “spatial registration of NIRS channel locations” function of the NIRSite 2020.7 software was used. The optodes were placed on the scalp using an elastic cap. Two detector optodes are placed at a distance of 30 mm from the source optodes. Rays of two different wavelengths were sent from the source optodes, 760 and 850 nm, and the rays were detected by the detector. HbO_2_ signals were used in the analysis, as HbO_2_ is a more reliable indicator of cortical activation (Dravida et al., 2018). Statistical analysis of the hemodynamic response functions (HRFs) of the obtained HbO_2_ cortical activation was measured.

### 2.4 Statistical analysis

Oxygenated hemoglobin concentration changes were recorded with the NIRStar Acquisition software, and the data were analyzed with the MATLAB‐based HOMER‐3 program (The MathWorks). A band pass filter with a low cut‐off frequency of 0 Hz and a high cut‐off frequency of 0.5 Hz was applied to the raw data to delete cardiac, respiratory, motor noise, and unwanted time series. To correct the motion artifacts of the participants during the experiment, the “motion correct wavelet” process was applied. For the experimental setup, HRF between 0 and 20 s after the stimulus was calculated, and between −2 and 0 s were evaluated for based HRF values.

SPSS version 26.0 software (SPSS Inc.) was used for statistical analysis of oxyhemoglobin concentration values calculated as a result of HOMER‐3 analysis. The suitability of the data sets obtained in the HRF evaluation to normal distribution was examined with the Kolmogorov–Smirnov Test. As a result of the analysis, it was determined that the data showed a normal distribution and Independent Sample *t*‐test was used to determine the significance between the HRF values between the groups. Mann–Whitney U test was used to determine the difference in age, educational status, and MoCA test score between the groups. *p* < .05 was determined as significance level.

## 3 RESULTS

### 3.1 Behavioral performance

There was no significant difference between the study and control groups in terms of age, gender, MoCA scores, and total number of targets specified in the auditory oddball task (Table 2).

**TABLE 2:** Demographic profiles of the participants.

VARIABLE	Control group	Study group	*p*
**Gender (male/female)**	3/1	0/3	.066
**Age (mean ± SD)**	30.5 ± 10.4	24.3 ± 1.1	.271
**MoCA scores (mean ± SD)**	29.5 ± 0.57	29.3 ± 0.57	.683

### 3.2 fNIRS analyses

Hemodynamic recordings were taken from a total of 45‐channel optodes placed on the frontal and temporal cortex during the auditory oddball task.

- When the HRF of HbO was compared between the groups before the auditory task, no significant difference was observed in any of the channels (*p* > .05).
- When the HRF of HbO was compared between the groups after the auditory task, a significant difference was observed in channels 1, 4–6, 9, 12–15, 17, 21, 23–27, 29, 32, 33, 35–38, 40–43, and 44 (*p* < .05).
- When the before and after auditory task HRFs of HbO were compared in the control group, a significant difference was observed in channels 5, 8, 11, 14, 17–22, 26, 28–31, 35, 36, 39, 41, 42, and 43 (*p* < .05).
- When the before and after auditory stimulus HRFs of HbO were compared in the study group, a significant difference was observed in channels 1, 4–16, 20–33, 35–43, and 44 (*p* < .05). The mean and standard deviation information of these comparisons are shown in Table 3.

**TABLE 3:** Comparison of hemodynamic response function (HRF) HbO changes between groups before/after auditory task.

*fNIRS* comparisons	HRF of HbO (Mean ± SD)	*p*
(i) Before the task **Control vs. study group**	3.24 E + 04 ± 1.84 E + 06 vs. 8.17 E + 03 ± 1.85 E + 06	>.05
(ii) After the task **control vs. study group**	2.29 E + 05 ± 3.55 E + 06 vs. 6.96 E + 05 ± 5.29 E + 06	<.05
(iii) Control group **Before vs. after the task**	3.24 E + 04 ± 1.84 E + 06 vs. 2.29 E + 05 ± 3.55 E + 06	<.05
(iv) Study group **Before vs. after the task**	8.17 E + 03 ± 1.85 E + 06 vs. 6.96 E + 05 ± 5.29 E + 06	<.05

*Note*: E + 0x means “extra count by x times.”

**FIGURE 3:**
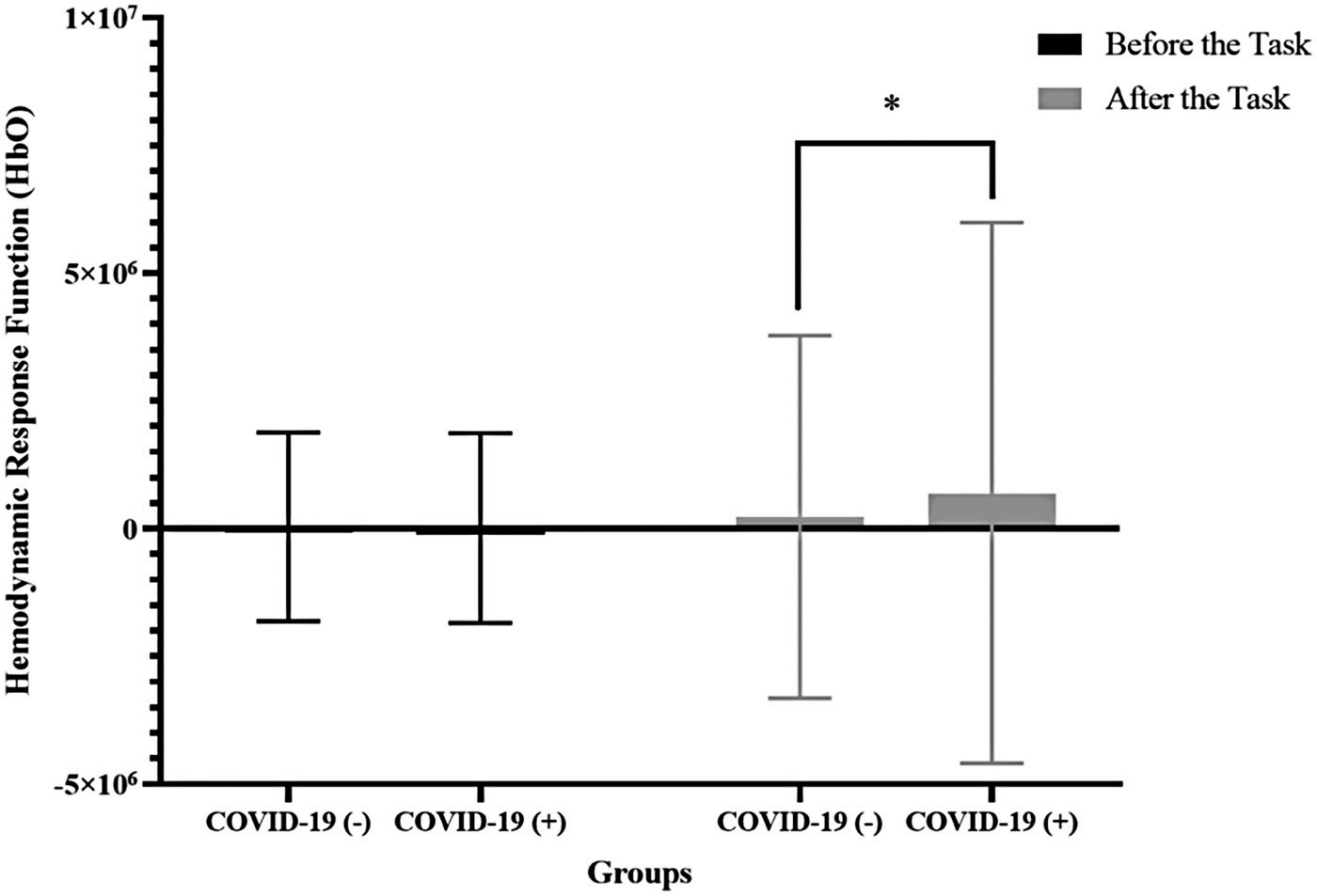
Hemodynamic response functions differences between groups in the before and after the task (*p < .05).

**FIGURE 4:**
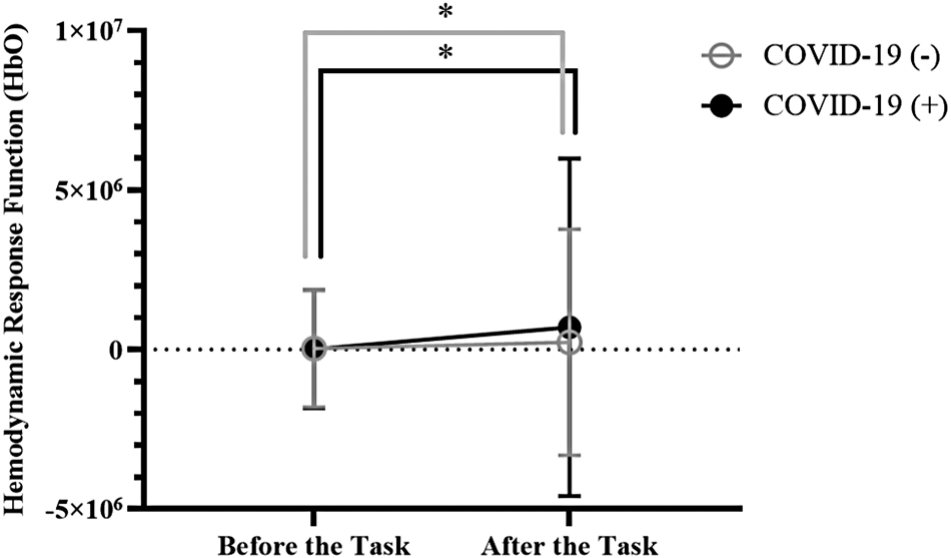
Hemodynamic response function differences between the tasks in the groups (*p < .05).

When the HRFs of HbO values measured before the auditory task were compared, there was no significant difference between the groups (*p* > .05). However, after the auditory task was initiated, a significant difference was observed between the groups (Figure 3). A significant increase in HRF of HbO was observed in both groups after the auditory task. This increase was higher in the study group (Table 3) (Figure 4).

**FIGURE 5:**
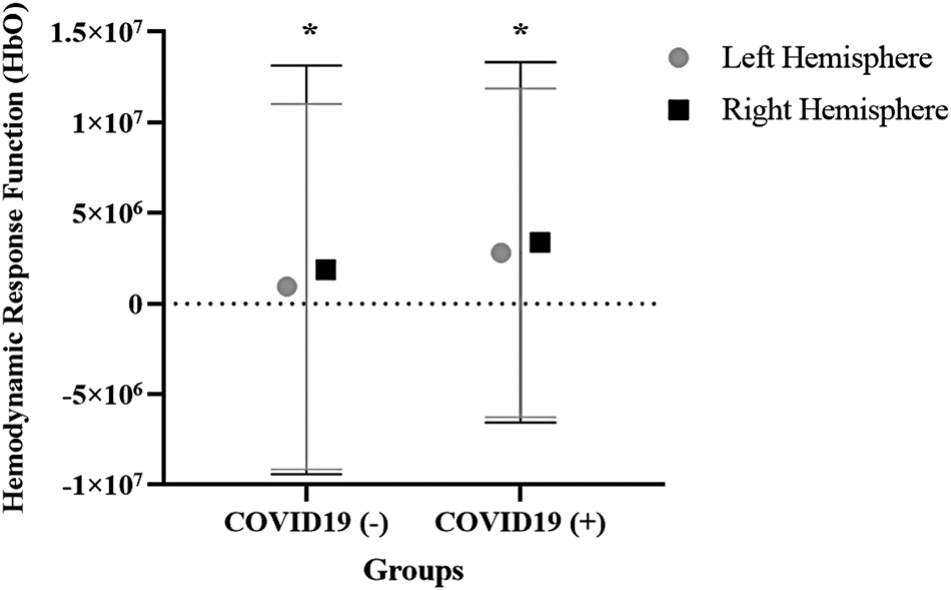
Hemodynamic activation changes in the right and left hemispheres after the auditory stimuli (*p < .05).

Hemodynamic activation changes of the right and left hemispheres were not significantly different before the auditory task in both groups. However, after the task, it was shown that the hemodynamic activation change in the right hemisphere was significantly higher compared to left hemisphere in both groups, which was especially higher in the study group (*p* < .05) (Figure 5).

**FIGURE 6:**
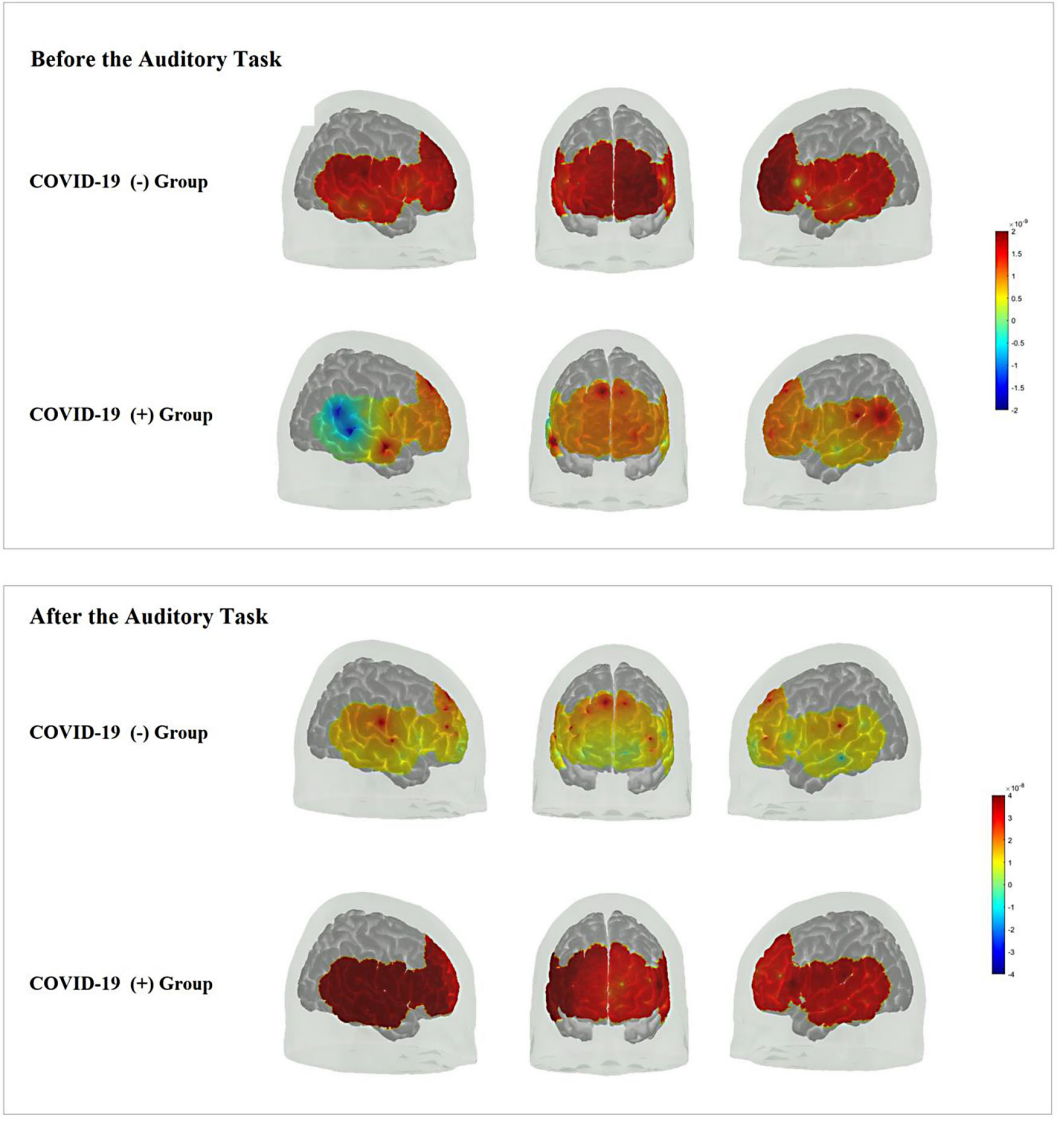
Representation and comparison of oxygenated hemodynamic response function (HRF) in micromolar unit with brain activations before and after auditory task (from left to right, right temporal region, frontal region, and left temporal region, respectively). It is important to consider the colormap values on the right; although the values are 10^−9^ in the before the auditory task visual, these are expressed as 10^−8^ in the after the auditory task. It is stated here that there is a more increase in HRF value after the task in the COVID‐19 (+) group, and this is observed more in the right hemisphere.

When the HbO activation of the groups were compared before and after the auditory stimulus, an increased HbO activation was observed in the left and right vmPFC regions of the frontal lobe as well as temporal lobe in the study group (Figure 6).

## 4 DISCUSSION

This study revealed the brain hemodynamic activation change during auditory attention skills of young people who had SARS‐CoV‐2 infection. Our results showed that during the task, significantly increased oxygenated brain hemodynamic activation responses were obtained in the subjects who had SARS‐CoV‐2 infection when compared to controls. There was significantly higher hemodynamic activation in the vmPFC region of the frontal lobe and superior temporal gyrus (STG) and medial temporal gyrus (MTG) brain regions of the temporal lobes bilaterally in the study group. In addition, significant hemodynamic activation differences were observed between the cerebral hemispheres in the study and control groups during the auditory attention task, and this activation was higher in the right hemisphere as well as in the study group. It should also be noted that, no significant difference was detected in the results of the MoCA test, in which we tested whether there were differences between groups in terms of cognitive functions. Therefore, increased HRF in the temporal and frontal regions in the study group may be a possible result of compensating for attention and working memory deficits in the study group.

Studies using the oddball paradigm on healthy participants have revealed significant differences in HRF to the target and nontarget auditory stimuli in different parts of the brain. fMRI and EEG neuroimaging methods to show brain regions involved in auditory target detection have shown activation in bilateral inferior frontal gyrus, dorsolateral prefrontal cortex, ventrolateral prefrontal cortex, inferior parietal lobe, inferior, middle and STG, supramarginal gyrus, insula, cingulate gyrus, and hippocampus (Halgren et al., 1998; Kiehl et al., 2001). An fMRI study in which the consistencies of the auditory oddball paradigm task and the related task in the brain region were investigated in healthy subjects revealed that significant hemodynamic activation was observed in the bilateral lateral frontal cortex and bilateral inferior parietal cortex (Kiehl & Liddle, 2003). As parallel with the previous studies, our study also revealed hemodynamic activation in the frontal and bilateral temporal lobes in both of the study and control groups following the analyses of cognitive auditory stimuli. The resulting hemodynamic activation was higher in the study group. These data are consistent with Sonkaya's study, which found that the basal blood flow rates after SARS‐CoV‐2 infection were higher than the healthy controls (Sonkaya et al., 2021). Previous studies performed by using different neuroimaging methods and biomarkers revealed cognitive deficits in SARS‐CoV‐2 infections as well (Akıncı et al., 2022).

It was reported that neurological symptoms like seizures and MRI perfusion abnormalities could be observed in the majority of individuals after SARS‐CoV‐2 infection, and these might be associated with hypoxic‐ischemic encephalopathy (Chougar et al., 2020). One of the most common findings in the acute phase of the SARS‐CoV‐2 infection can be the presence of rhythmic delta waves in the frontal lobes, irregularity in background activity, and epileptiform discharges (Antony & Haneef, 2020; Santos De Lima et al., 2021; Vespignani et al., 2020). However, in some cases, hypoperfusion can also be observed in the frontal and temporal lobes (Lambrecq et al., 2021; Zhan et al., 2020; Patient Led Research Collaborative 2022; Helms et al., 2020; Galanopoulou et al., 2020), and it has been shown that nonhospitalized patients have detrimental effects on brain structures such as impairments in olfactory‐related regions, the functionally connected areas to the temporal piriform cortex, and a diffusion index in the superior fronto‐occipital fasciculus (Douaud et al., 2022). fMRI studies have shown a widespread reduction in greater gray matter thickness in the frontoparietal and temporal regions of patients with SASRS‐CoV‐2 infection, which may secondarily affect the frontal–temporal network due to increased body temperature or lack of oxygen during the disease (Duan et al., 2021; Abdallah, 2021). In line with these findings, our current study found that significantly increased HRF in the vmPFC region of the frontal lobe and STG and MTG brain regions in the bilateral temporal lobe in the study group during auditory attention. This finding is consistent with the central auditory processing disorder result consistent which evaluated auditory attention in a patient with normal hearing following SARS‐CoV‐2 infection (Andreeva et al., 2022). The vmPFC regions are associated with higher working memory performance during working memory retention phase, and vmPFC is associated with higher working memory performance during encoding phase (Mukahirwa et al., 2021; Krawczyk, 2002); moreover, there are neuroimaging studies showing that STG has a strong connection with speech flow in the presence of background noise, which has a significant contribution to cognitive function (Stevens et al., 2000), and that MTG, which has functions such as voice recognition, language processing, understandable speech, and verbal mental arithmetic processing, is active during auditory target detection (Stevens et al., 2005; Kiehl et al., 2005; Xu et al., 2015). Considering the cognitive and auditory functions of vmPFC, STG, and MTG, together with the findings obtained in our study, it can be thought that SARS‐CoV‐2 infection has an effect on hearing‐related attention mechanisms.

Functional asymmetries are neural activities that are stronger in one cerebral hemisphere than in the other. The evidence from neuroimaging studies suggests that the right hemisphere of the human brain may be more specialized for attention than the left hemisphere. According to fMRI studies, in which the activities of hemispheres were investigated in the auditory oddball paradigm, a greater activity could be detected in the right hemisphere compared with the activities in the left anterior, temporal, and parietal lobes (Stevens et al., 2005). These results are also consistent with the studies in which the oddball paradigm effect on event‐related potentials and mismatch negativity with electroencephalography was investigated (Alexander et al., 1996; Oades et al., 1995; Rinne et al., 2000; Jemel et al., 2002). In one of the studies in which the subjects with SARS‐CoV‐2 infection and healthy controls were compared regarding the right and left hemispheres imaging‐derived phenotypes, no significant reductions could be found in the gray matter thickness of the hemispheres in the infected patients (Douaud et al., 2022). In our study, in which we also examined attention‐related neural activity to the rare frequency auditory stimulus, the right hemisphere activation was significantly higher in both study and control groups. In line with our findings, one of the MRI studies which investigated the microstructural changes in the central nervous system after infection showed that the spreading abnormalities in the white matter were confined to the right hemisphere in the absence of asymmetric symptoms reported by the patients (Lu et al., 2020). These findings support the contention of improved compensation for the hearing‐related attention mechanisms of the patients who had SARS‐CoV‐2 infection.

It is plausible to say that the increased neuronal activity may indicate a higher mental effort while performing auditory tasks which require concentration and attention resources and subsequently may lead to fatigue. In this study population, the hearing was evaluated based on the participant's statement alone, unfortunately. The assessment of hearing and central auditory processing with objective and subjective test batteries in similar groups will contribute to understanding the effects of COVID‐19.

A limitation of our study is that we could not have the pre‐disease cognitive assessment results of the participants in the COVID‐19 (+) group. Besides, the sample group had to be limited due to time constraints, access to experimental group individuals, and changes in the variant of the disease during the COVID‐19 pandemic process. However, we believe that despite these disabilities, our study will provide insight on evaluating cognitive findings and auditory performance together within the scope of COVID‐19 studies.

## 5 CONCLUSION

The difficulty in hearing orientation is one of the important features of central disorders, and SARS‐CoV‐2 infection may impact on central auditory processing or auditory attention due to changes in oxyhemoglobin levels in the temporal brain regions. It seems that SARS‐CoV‐2 infection is associated with an additional load on the neural activity, and difficulties in focusing in auditory attention, following speech and hearing in noise as well as increased effort to perceive auditory cues. These factors may result in fatigue and poor performance in daily life.

## AUTHOR CONTRIBUTIONS

**Handan Yaman**: Conceptualization; methodology; data collection and analysis; interpretation of data; visualization; formal analysis; writing—review and editing. **Oğuz Yılmaz**: Conceptualization; methodology; interpretation of data; investigation; supervision; validation; writing—review and editing. **Lütfü Hanoğlu**: Conceptualization; methodology; interpretation of data; investigation; supervision; validation; writing—review and editing. **Yıldırım Bayazıt**: Conceptualization; methodology; interpretation of data; investigation; supervision; validation; writing—review and editing;.

## CONFLICT OF INTEREST STATEMENT

The authors declare that they have no conflicts of interest.

## FUNDING INFORMATION

No external funding was provided for this study.

### PEER REVIEW

The peer review history for this article is available at https://publons.com/publon/10.1002/brb3.3303.

## Data Availability

The data used to support the findings of this study are included within the article.

## References

[brb33303-bib-0038] Abdallah, C. G. (2021). Brain Networks Associated With COVID‐19 Risk: Data From 3662 Participants. Chronic Stress, 5, 24705470211066770. 10.1177/24705470211066770/ASSET/IMAGES/LARGE/10.1177_24705470211066770-FIG2.JPEG 34993375 PMC8725219

[brb33303-bib-0026] Akıncı, B. , Oğul, O. E. , Hanoğlu, L. , Kulaç, B. , Ören, D. , Ulu, O. , & Basançelebi, B. (2022). Evaluation of cognitive functions in adult individuals with COVID‐19. Neurological Sciences, 1, 1–10. 10.1007/s10072-022-06562-2 PMC9793347 36574178

[brb33303-bib-0047] Alexander, J. E. , Bauer, L. O. , Kuperman, S. , Morzorati, S. , O'Connor, S. J. , Rohrbaugh, J. , Porjesz, B. , Begleiter, H. , & Polich, J. (1996). Hemispheric differences for P300 amplitude from an auditory oddball task. International Journal of Psychophysiology, 21(2–3), 189–196. 10.1016/0167-8760(95)00047-X 8792206

[brb33303-bib-0039] Andreeva, I. G. , Gvozdeva, A. , Pimenova, V. , Ryabkova, V. , Lukashenko, M. , Kamaeva, E. , Shapkina, V. , Soprun, L. , Gavrilova, N. , Fedotkina, T. V. , Churilov, L. P. , & Shoenfeld, Y. (2022). Assessment of hearing and vestibular functions in a post‐COVID‐19 patient: A clinical case study. Diagnostics, 13(1), 122. 10.3390/diagnostics13010122 36611414 PMC9819003

[brb33303-bib-0029] Antony, A. R. , & Haneef, Z. (2020). Systematic review of EEG findings in 617 patients diagnosed with COVID‐19. Seizure, 83, 234. 10.1016/j.seizure.2020.10.014 33121875 PMC7569418

[brb33303-bib-0015] Calmels, M. N. , Gallois, Y. , Marx, M. , Deguine, O. , Taoui, S. , Arnaud, E. , Strelnikov, K. , & Barone, P. (2022). Functional reorganization of the central auditory system in children with single‐sided deafness: A protocol using fNIRS. Brain Sciences, 12(4), 423. 10.3390/brainsci12040423 35447955 PMC9029510

[brb33303-bib-0007] Carod‐Artal, F. J. (2021). Post‐COVID‐19 syndrome: Epidemiology, diagnostic criteria and pathogenic mechanisms involved. Revue Neurologique, 72(11), 384–396. 10.33588/rn.7211.2021230 34042167

[brb33303-bib-0008] Ceban, F. , Ling, S. , Lui, L. M. W. , Lee, Y. , Gill, H. , Teopiz, K. M. , Rodrigues, N. B. , Subramaniapillai, M. , Di Vincenzo, J. D. , Cao, B. , Lin, K. , Mansur, R. B. , Ho, R. C. , Rosenblat, J. D. , Miskowiak, K. W. , Vinberg, M. , Maletic, V. , & McIntyre, R. S. (2022). Fatigue and cognitive impairment in post‐COVID‐19 syndrome: A systematic review and meta‐analysis. Brain, Behavior, and Immunity, 101, 93–135. 10.1016/j.bbi.2021.12.020 34973396 PMC8715665

[brb33303-bib-0027] Chougar, L. , Shor, N. , Weiss, N. , Galanaud, D. , Leclercq, D. , Mathon, B. , Belkacem, S. , Ströer, S. , Burrel, S. , Boutolleau, D. , Demoule, A. , Rosso, C. , Delorme, C. , Seilhean, D. , Dormont, D. , Morawiec, E. , Raux, M. , Demeret, S. , Gerber, S. , … CoCo Neurosciences Study Group . Retrospective observational study of brain MRI findings in patients with acute SARS‐CoV‐2 infection and neurologic manifestations. Radiology, 297(3), E313–E323. 10.1148/radiol.2020202422 PMC7370354 32677875

[brb33303-bib-0020] Cope, M. , Delpy, D. T. , Reynolds, E. O. , Wray, S. , Wyatt, J. , & van der Zee, P. (1988). Methods of quantitating cerebral near infrared spectroscopy data. Advances in Experimental Medicine and Biology, 222, 183–189. 10.1007/978-1-4615-9510-6_21 3129910

[brb33303-bib-0004] Davis, H. E. , Assaf, G. S. , McCorkell, L. , Wei, H. , Low, R. J. , Re'em, Y. , Redfield, S. , Austin, J. P. , & Akrami, A. (2021). Characterizing long COVID in an international cohort: 7 months of symptoms and their impact. EClinicalMedicine, 38, 101019. 10.1016/j.eclinm.2021.101019 34308300 PMC8280690

[brb33303-bib-0036] Douaud, G. , Lee, S. , Alfaro‐Almagro, F. , Arthofer, C. , Wang, C. , McCarthy, P. , Lange, F. , Andersson, J. L. R. , Griffanti, L. , Duff, E. , Jbabdi, S. , Taschler, B. , Keating, P. , Winkler, A. M. , Collins, R. , Matthews, P. M. , Allen, N. , Miller, K. L. , Nichols, T. E. , & Smith, S. M. (2022). SARS‐CoV‐2 is associated with changes in brain structure in UK Biobank. Nature, 604(7907), 697–707. 10.1038/s41586-022-04569-5 35255491 PMC9046077

[brb33303-bib-0021] Dravida, S. , Noah, J. A. , Zhang, X. , & Hirsch, J. (2018). Comparison of oxyhemoglobin and deoxyhemoglobin signal reliability with and without global mean removal for digit manipulation motor tasks. Neurophotonics, 5(1), 011006. 10.1117/1.NPh.5.1.011006 28924566 PMC5597778

[brb33303-bib-0037] Duan, K. , Premi, E. , Pilotto, A. , Cristillo, V. , Benussi, A. , Libri, I. , Giunta, M. , Bockholt, H. J. , Liu, J. , Campora, R. , Pezzini, A. , Gasparotti, R. , Magoni, M. , Padovani, A. , & Calhoun, V. D. (2021). Alterations of frontal‐temporal gray matter volume associate with clinical measures of older adults with COVID‐19. Neurobiology of Stress, 14, 100326. 10.1016/j.ynstr.2021.100326 33869679 PMC8041745

[brb33303-bib-0035] Galanopoulou, A. S. , Ferastraoaru, V. , Correa, D. J. , Cherian, K. , Duberstein, S. , Gursky, J. , Hanumanthu, R. , Hung, C. , Molinero, I. , Khodakivska, O. , Legatt, A. D. , Patel, P. , Rosengard, J. , Rubens, E. , Sugrue, W. , Yozawitz, E. , Mehler, M. F. , Ballaban‐Gil, K. , Haut, S. R. , … Boro, A. (2020). EEG findings in acutely ill patients investigated for SARS‐CoV‐2/COVID‐19: A small case series preliminary report. Epilepsia Open, 5(2), 314–324. 10.1002/epi4.12399 32537529 PMC7289172

[brb33303-bib-0023] Halgren, E. , Marinkovic, K. , & Chauvel, P. (1998). Generators of the late cognitive potentials in auditory and visual oddball tasks. Electroencephalography and Clinical Neurophysiology, 106(2), 156–164. 10.1016/S0013-4694(97)00119-3 9741777

[brb33303-bib-0009] Hampshire, A. , Trender, W. , Chamberlain, S. R. , Jolly, A. E. , Grant, J. E. , Patrick, F. , Mazibuko, N. , Williams, S. C. , Barnby, J. M. , Hellyer, P. , & Mehta, M. A. (2021). Cognitive deficits in people who have recovered from COVID‐19. EClinicalMedicine, 39, 101044. 10.1016/j.eclinm.2021.101044 34316551 PMC8298139

[brb33303-bib-0034] Helms, J. , Kremer, S. , Merdji, H. , Clere‐Jehl, R. , Schenck, M. , Kummerlen, C. , Collange, O. , Boulay, C. , Fafi‐Kremer, S. , Ohana, M. , Anheim, M. , & Meziani, F. (2020). Neurologic features in severe SARS‐CoV‐2 infection. New England Journal of Medicine, 382(23), 2268–2270. 32294339 10.1056/NEJMc2008597 PMC7179967

[brb33303-bib-0001] Huang, C. , Huang, L. , Wang, Y. , Li, X. , Ren, L. , Gu, X. , Kang, L. , Guo, L. , Liu, M. , Zhou, X. , Luo, J. , Huang, Z. , Tu, S. , Zhao, Y. , Chen, L. , Xu, D. , Li, Y. , Li, C. , Peng, L. , … Cao, B. (2021). 6‐Month consequences of COVID‐19 in patients discharged from hospital: A cohort study. The Lancet, 397(10270), 220–232. 10.1016/S0140-6736(20)32656-8/ATTACHMENT/2A67FD00-0525-4528-B4ED-944C31313F8C/MMC1.PDF PMC7833295 33428867

[brb33303-bib-0010] Jafari, Z. , Kolb, B. E. , & Mohajerani, M. H. (2022). Hearing loss, tinnitus, and dizziness in COVID‐19: A systematic review and meta‐analysis. Canadian Journal of Neurological Sciences, 49(2), 184–195. 10.1017/cjn.2021.63 PMC8267343 33843530

[brb33303-bib-0050] Jemel, B. , Achenbach, C. , Müller, B. W. , Röpcke, B. , & Oades, R. D. (2002). Mismatch negativity results from bilateral asymmetric dipole sources in the frontal and temporal lobes. Brain Topography, 15(1), 13–27. 10.1023/A:1019944805499 12371672

[brb33303-bib-0018] Kang, J. M. , Cho, Y. S. , Park, S. , Lee, B. H. , Sohn, B. K. , Choi, C. H. , Choi, J. S. , Jeong, H. Y. , Cho, S. J. , Lee, J. H. , & Lee, J. Y. (2018). Montreal cognitive assessment reflects cognitive reserve. BMC Geriatrics, 18(1), 261. 10.1186/s12877-018-0951-8 30376815 PMC6208087

[brb33303-bib-0024] Kiehl, K. A. , & Liddle, P. F. (2003). Reproducibility of the hemodynamic response to auditory oddball stimuli: A six‐week test‐retest study. Human Brain Mapping, 18(1), 42–52. 10.1002/hbm.10074 12454911 PMC6872095

[brb33303-bib-0022] Kiehl, K. A. , Laurens, K. R. , Duty, T. L. , Forster, B. B. , & Liddle, P. F. (2001). Neural sources involved in auditory target detection and novelty processing: An event‐related fMRI study. Psychophysiology, 38(1), 133–142. 10.1111/1469-8986.3810133 11321614

[brb33303-bib-0044] Kiehl, K. A. , Stevens, M. C. , Laurens, K. R. , Pearlson, G. , Calhoun, V. D. , & Liddle, P. F. (2005). An adaptive reflexive processing model of neurocognitive function: Supporting evidence from a large scale (*n* = 100) fMRI study of an auditory oddball task. Neuroimage, 25(3), 899–915. 10.1016/j.neuroimage.2004.12.035 15808990

[brb33303-bib-0041] Krawczyk, D. C. (2002). Contributions of the prefrontal cortex to the neural basis of human decision making. Neuroscience & Biobehavioral Reviews, 26(6), 631–664. 10.1016/S0149-7634(02)00021-0 12479840

[brb33303-bib-0031] Lambrecq, V. , Hanin, A. , Munoz‐Musat, E. , Chougar, L. , Gassama, S. , Delorme, C. , Cousyn, L. , Borden, A. , Damiano, M. , Frazzini, V. , Huberfeld, G. , Landgraf, F. , Nguyen‐Michel, V. H. , Pichit, P. , Sangare, A. , Chavez, M. , Morélot‐Panzini, C. , Morawiec, E. , Raux, M. , … Cohort COVID‐19 Neurosciences (CoCo Neurosciences) Study Group . Association of clinical, biological, and brain magnetic resonance imaging findings with electroencephalographic findings for patients with COVID‐19. JAMA Network Open, 4(3), e211489. 10.1001/jamanetworkopen.2021.1489 PMC7961310 33720371

[brb33303-bib-0051] Lu, Y. , Li, X. , Geng, D. , Mei, N. , Wu, P. Y. , Huang, C. C. , Jia, T. , Zhao, Y. , Wang, D. , Xiao, A. , & Yin, B. (2020). Cerebral micro‐structural changes in COVID‐19 patients—An MRI‐based 3‐month follow‐up study. EClinicalMedicine, 25, 100484. 10.1016/j.eclinm.2020.100484 32838240 PMC7396952

[brb33303-bib-0019] Mahendran, R. , Chua, J. , Feng, L. , Kua, E. H. , & Preedy, V. R. (2015). The mini‐mental state examination and other neuropsychological assessment tools for detecting cognitive decline. In, Diet and nutrition in dementia and cognitive decline (pp. 1159–1174). Academic Press. 10.1016/B978-0-12-407824-6.00109-9

[brb33303-bib-0005] Marshall, M. (2020). The lasting misery of coronavirus long‐haulers. Nature, 585(7825), 339–341. 10.1038/d41586-020-02598-6 32929257

[brb33303-bib-0017] McLinden, J. , Borgheai, S. B. , Hosni, S. , Kumar, C. , Rahimi, N. , Shao, M. , Spencer, K. M. , & Shahriari, Y. (2023). Individual‐specific characterization of event‐related hemodynamic responses during an auditory task: An exploratory study. Behavioural Brain Research, 436, 114074. 10.1016/j.bbr.2022.114074 36028001

[brb33303-bib-0040] Mukahirwa, J. , Eun, S. , Kang, M. , Yoon, T. , & Park, K. (2021). Deactivation of the attention‐shifting ventromedial prefrontal cortex during the encoding and hold phases predicts working memory performance. NeuroReport, 32(18), 1408–1415. 10.1097/WNR.0000000000001744 34743168

[brb33303-bib-0012] Mustafa, M. W. M. (2020). Audiological profile of asymptomatic Covid‐19 PCR‐positive cases. American Journal of Otolaryngology, 41(3), 102483. 10.1016/j.amjoto.2020.102483 32307189 PMC7151386

[brb33303-bib-0048] Oades, R. D. , Zerbin, D. , & Dittmann‐Balcar, A. (1995). The topography of event‐related potentials in passive and active conditions of a 3‐tone auditory oddball test. International Journal of Neuroscience, 81(3–4), 249–264. 10.3109/00207459509004890 7628914

[brb33303-bib-0006] Patient Led Research Collaborative . (2022). Report: What does COVID‐19 recovery actually look like? Patient Led Research Collaborative. Accessed August 7, 2022. https://patientresearchcovid19.com/research/report‐1/

[brb33303-bib-0049] Rinne, T. , Alho, K. , Ilmoniemi, R. J. , Virtanen, J. , & Näätänen, R. (2000). Separate time behaviors of the temporal and frontal mismatch negativity sources. Neuroimage, 12(1), 14–19. 10.1006/nimg.2000.0591 10875898

[brb33303-bib-0030] Santos De Lima, F. , Issa, N. , Seibert, K. , Davis, J. , Wlodarski, R. , Klein, S. , El Ammar, F. , Wu, S. , Rose, S. , Warnke, P. , & Tao, J. (2021). Epileptiform activity and seizures in patients with COVID‐19. Journal of Neurology, Neurosurgery, and Psychiatry, 92(5), 565–566. 10.1136/jnnp-2020-324337 33158913

[brb33303-bib-0033] Sarikaya, B. (2020). More on neurologic features in severe SARS‐CoV‐2 infection. The New England Journal of Medicine, 382(26), e110. 10.1056/NEJMc2015132 32453516

[brb33303-bib-0025] Sonkaya, A. R. , Öztürk, B. , & Karadaş, Ö. (2021). Cerebral hemodynamic alterations in patients with COVID‐19. Turkish Journal of Medical Sciences, 51(2), 435–439. 10.3906/sag-2006-203 33021761 PMC8203147

[brb33303-bib-0003] Soriano, J. B. , Murthy, S. , Marshall, J. C. , Relan, P. , & Diaz, J. V. (2022). A clinical case definition of post‐COVID‐19 condition by a Delphi consensus. The Lancet Infectious Diseases, 22(4), e102–e107. 10.1016/S1473-3099(21)00703-9 34951953 PMC8691845

[brb33303-bib-0013] Sriwijitalai, W. , & Wiwanitkit, V. (2020). Hearing loss and COVID‐19: A note. American Journal of Otolaryngology—Head and Neck Medicine and Surgery, 41(3), 102473. 10.1016/j.amjoto.2020.102473 PMC7132500 32276732

[brb33303-bib-0042] Stevens, A. A. , Skudlarski, P. , Gatenby, J. C. , & Gore, J. C. (2000). Event‐related fMRI of auditory and visual oddball tasks. Magnetic Resonance Imaging, 18(5), 495–502. 10.1016/S0730-725X(00)00128-4 10913710

[brb33303-bib-0043] Stevens, M. C. , Calhoun, V. D. , & Kiehl, K. A. (2005). Hemispheric differences in hemodynamics elicited by auditory oddball stimuli. Neuroimage, 26(3), 782–792. 10.1016/j.neuroimage.2005.02.044 15955488 PMC2759643

[brb33303-bib-0046] Stevens, M. C. , Calhoun, V. D. , & Kiehl, K. A. (2005). Hemispheric differences in hemodynamics elicited by auditory oddball stimuli. Neuroimage, 26(3), 782. 10.1016/j.neuroimage.2005.02.044 15955488 PMC2759643

[brb33303-bib-0014] Strait, M. , & Scheutz, M. (2014). What we can and cannot (yet) do with functional near infrared spectroscopy. Frontiers in Neuroscience, 8, 117. 10.3389/FNINS.2014.00117/XML/NLM 24904261 PMC4033094

[brb33303-bib-0002] Tenforde, M. W. , Kim, S. S. , Lindsell, C. J. , Billig Rose, E. , Shapiro, N. I. , Files, D. C. , Gibbs, K. W. , Erickson, H. L. , Steingrub, J. S. , Smithline, H. A. , Gong, M. N. , Aboodi, M. S. , Exline, M. C. , Henning, D. J. , Wilson, J. G. , Khan, A. , Qadir, N. , Brown, S. M. , Peltan, I. D. , … IVY Network Investigators . (2022). Symptom duration and risk factors for delayed return to usual health among outpatients with COVID‐19 in a multistate health care systems network — United States, March–June 2020. Morbidity and Mortality Weekly Report, 69(30), 993–998. 10.15585/mmwr.mm6930e1 PMC7392393 32730238

[brb33303-bib-0011] Trecca, E. M. C. , Gelardi, M. , & Cassano, M. (2020). COVID‐19 and hearing difficulties. American Journal of Otolaryngology, 41(4), 102496. 10.1016/j.amjoto.2020.102496 32327217 PMC7166300

[brb33303-bib-0028] Vespignani, H. , Colas, D. , Lavin, B. S. , Soufflet, C. , Maillard, L. , Pourcher, V. , Paccoud, O. , Medjebar, S. , & Frouin, P. Y. (2020). Report on electroencephalographic findings in critically Ill patients with COVID‐19. Annals of Neurology, 88(3), 626. 10.1002/ana.25814 32533727 PMC7323170

[brb33303-bib-0045] Xu, J. , Wang, J. , Fan, L. , Li, H. , Zhang, W. , Hu, Q. , & Jiang, T. (2015). Tractography‐based Parcellation of the Human Middle Temporal Gyrus. Scientific Reports, 5, 18883. 10.1038/srep18883 26689815 PMC4686935

[brb33303-bib-0016] Zaramella, P. , Freato, F. , Amigoni, A. , Salvadori, S. , Marangoni, P. , Suppiej, A. , Schiavo, B. , & Chiandetti, L. (2001). Brain auditory activation measured by near‐infrared spectroscopy (NIRS) in neonates. Pediatric Research, 49(2), 213–219. 10.1203/00006450-200102000-00014 11158516

[brb33303-bib-0032] Zhan, M. , Qin, Y. , Xue, X. , & Zhu, S. (2020). Death from COVID‐19 of 23 health care workers in China. The New England Journal of Medicine, 382(23), 2267–2268. 10.1056/NEJMc2005696 32294342 PMC7179960

